# Gasdermin D membrane pores orchestrate IL-1α secretion from necrotic macrophages after NFS-rich silica exposure

**DOI:** 10.1007/s00204-023-03463-x

**Published:** 2023-02-25

**Authors:** Riccardo Leinardi, Amandine Pochet, Francine Uwambayinema, Yousof Yakoub, Valérie Quesniaux, Bernhard Ryffel, Petr Broz, Cristina Pavan, François Huaux

**Affiliations:** 1 Louvain Centre for Toxicology and Applied Pharmacology (LTAP), Institut de Recherche Expérimentale et Clinique (IREC), Université Catholique de Louvain, Brussels, Belgium; 2 Laboratory of Experimental and Molecular Immunology and Neurogenetics (INEM), UMR 7355 CNRS, University of Orleans and Artimmune, Orléans, France; 3 Department of Immunobiology, University of Lausanne, Epalinges, Switzerland; 4 Department of Chemistry, “G. Scansetti” Interdepartmental Center for Studies On Asbestos and Other Toxic Particulates, University of Torino, Torino, Italy

**Keywords:** IL-1α, Silica cytotoxicity, Macrophages, Gasdermin D pores, Necrosis, Silanols

## Abstract

IL-1α is an intracellular danger signal (DAMP) released by macrophages contributing to the development of silica-induced lung inflammation. The exact molecular mechanism orchestrating IL-1α extracellular release from particle-exposed macrophages is still unclear. To delineate this process, murine J774 and bone-marrow derived macrophages were exposed to increasing concentrations (1–40 cm^2^/ml) of a set of amorphous and crystalline silica particles with different surface chemical features. In particular, these characteristics include the content of nearly free silanols (NFS), a silanol population responsible for silica cytotoxicity recently identified. We first observed de novo stocks of IL-1α in macrophages after silica internalization regardless of particle physico-chemical characteristics and cell stress. IL-1α intracellular production and accumulation were observed by exposing macrophages to biologically-inert or cytotoxic crystalline and amorphous silicas. In contrast, only NFS-rich reactive silica particles triggered IL-1α release into the extracellular milieu from necrotic macrophages. We demonstrate that IL-1α is actively secreted through the formation of gasdermin D (GSDMD) pores in the plasma membrane and not passively released after macrophage plasma membrane lysis. Our findings indicate that the GSDMD pore-dependent secretion of IL-1α stock from macrophages solely depends on cytotoxicity induced by NFS-rich silica. This new regulated process represents a key first event in the mechanism of silica toxicity, suitable to refine the existing adverse outcome pathway (AOP) for predicting the inflammatory activity of silicas.

## Introduction

Interleukin 1-alpha (IL-1α) is a dual-function pro-inflammatory cytokine of the IL-1 family, and one of the leading molecules acting as a damage-associated molecular pattern (DAMP) (Dinarello 2018). IL-1α is involved in the induction of innate and adaptive immune responses, (auto-)inflammatory diseases and cancer development (Bertheloot and Latz 2017; Di Paolo and Shayakhmetov 2016; Dinarello 2011; Malik and Kanneganti 2018; Rider et al. 2013). IL-1α is synthesized as a pro-form (31 kDa), which is already active (Mosley et al. 1987). Pro-IL-1α may be cytosolic, membrane-bound, nuclear, or released into the extracellular environment, and possesses a N-terminal Nuclear Localization Sequence (NLS) that directs the protein to the nucleus after specific stimuli (Werman et al. 2004). In the nucleus, pro-IL-1α can regulate the activity of several transcriptional factors inducing the expression of a large portfolio of pro-inflammatory genes (i.e. pro-IL-1β, IL-6, and IL-8) (Dinarello 2018). The Ca^2+^-activated protease calpain, associated to the plasmatic cell membrane or in the cytosol of many cell types, including macrophages, canonically cleaves pro-IL-1α into the mature IL-1α form (17 kDa) after necrotic stimuli (Carruth et al. 1991; Kobayashi et al. 1990; Zheng et al. 2013). Both the precursor and mature IL-1α form bind the IL-1R1 receptor on the plasma membrane of tissue resident macrophages, promoting the transcription of inflammatory mediators via the NFkB pathway (Kono et al. 2014). After cleavage, the N-terminal pro-piece containing the NLS is removed increasing the affinity of IL-1α for the IL-1R1 receptor and reducing the ability IL-1α to translocate into the nucleus (Rider et al. 2013). Unlike most cytokines, which are up-regulated upon stimulation, IL-1α is constitutively present in resting cells under homeostatic conditions and released into the extracellular space following plasma membrane rupture from necrotic cells as a bioactive mediator (Dinarello 2009; Rider et al. 2013). Besides passive release, recent investigations showed that mature IL-1α is actively secreted by an organized process involving pore-forming proteins that regulates the secretion of mature IL-1 from pyroptotic macrophages (Evavold et al. 2018; Pyrillou et al. 2020; Tsuchiya et al. 2021).

Silica represents one of the most studied materials because of its wide applications and peculiar physicochemical properties which make it appealing for new nanotechnologies (Croissant et al. 2020; Jeelani et al. 2019). However, the prolonged exposure to respirable crystalline silica particles induces severe lung pathologies, including inflammation, silicosis, lung cancer, and systemic autoimmune diseases (IARC 2012; Leung et al. 2012). Moreover, in vivo studies recently pointed out lung toxic effects in response to some types of amorphous silica particles in animal models, in particular fumed silica (Croissant et al. 2020; Murugadoss et al. 2017; Rubio et al. 2019). It has been recently demonstrated that a specific population of surface hydroxyl groups, namely the “nearly free silanols” (NFS), which are variably present on both amorphous and crystalline silica particles, is critical in initiating inflammatory responses to silica by interacting with biomembranes (Pavan et al. 2020, 2022). Former research demonstrated that IL-1α is one of the leader cytokines in acute lung inflammation induced by silica (Rabolli et al. 2014; Skuland et al. 2020). Following crystalline silica instillation in mice, lung stocks of IL-1α, mostly contained in alveolar macrophages, were rapidly released in the alveolar space, triggering IL-1β secretion and neutrophil accumulation. We also observed that cultured murine J774 macrophages possessed high concentration of constitutive IL-1α representing a validated in vitro bioassay to assess IL-1α release and determine the inflammatory activity of particles (Rabolli et al. 2014). Although the key role of IL-1α in silica-induced lung inflammation is known, the exact mechanism orchestrating its release from silica-exposed macrophages remains unclear.

In the present work, cultured murine J774 cells and bone-marrow derived macrophages (BMDM) were exposed to silica to clarify the mechanism of IL-1α expression, intracellular accumulation, and extracellular release. We show the rapid increase of IL-1α intracellular content after silica internalization, which was followed by sustained extracellular secretion by macrophages. This process was modulated by GSDMD-induced pores, which were formed in the plasma membrane of necrotic macrophages in response to cytotoxic silica particles exposure. To understand the role of particle physico-chemical characteristics in triggering IL-1α release, we used a broad set of crystalline and amorphous silica particles with different NFS content. We show that only extracellular IL-1α secretion (not its intracellular accumulation) depends on the NFS-related surface chemistry of particles, which drives cytotoxicity, necrosis and, as a final outcome, plasma membrane pore formation. Our data provide insights into the mechanism regulating IL-1α availability during silica-induced inflammation.

## Materials and methods

### Silica samples

The set of silica samples and their physico-chemical properties are summarized in Table 1, and included: (cQ-f) a commercial fractured microcrystalline α-quartz (Min-U-Sil 5), largely used in studies of experimental silicosis and lung cancer [(IARC) 1997], purchased from U.S. Silica Co. (Berkeley Springs, WV, lot number 15062696); (mQ-f) a quartz dust obtained by grinding a very pure crystal from Madagascar in a planetary ball mill (Retsch S100, GmbH, Haan, Germany) for 3 h (70 rpm), then in a mixer mill (Retsch MM200) for 1 h (27 Hz) using agate jars; (sQ) synthetic highly pure quartz crystals in submicron size obtained by hydrothermal synthesis, following a procedure previously described (Pastero et al. 2016; Pavan et al. 2020); (A50) Aerosil OX 50, a fumed silica purchased from Degussa (Frankfurt A.M., Germany); and (VS) a vitreous silica obtained by grinding a very pure silica glass (Suprasil) in a ball mill (agate jar) for 3 h (70 rpm).

**Table 1:** Physico-chemical properties of the investigated crystalline and amorphous silica particles

Sample	Origin	Surface state	Particle Size (μm ± s.d.)	SSA^c^ (m^2^ g^−1^)	References
sQ	Synthetic	As-grown (crystalline)	1.3 ± 2.3^a^	5.8	Leinardi et al. (2020); Pavan et al. (2020); Turci et al. (2016)
cQ-f (Min-U-Sil 5)	Commercial	Fractured (crystalline)	1.0 ± 1.2^a^	4.3	Leinardi et al. (2020)
mQ-f	Natural	Fractured (crystalline)	1.7 ± 1.2^a^	3.8	Ghiazza et al. (2010); Pavan et al. (2014)
A50 (Aerosil 50)	Commercial	amorphous	0.04^b^	57	Pavan et al. (2020, 2013)
VS (Suprasil)	Commercial	amorphous, (fractured)	1.6 ± 1.2^a^	3.1	Ghiazza et al. (2010); Pavan et al. (2014)

^a^Measured by flow particle image analysis (FPIA) in ultrapure H_2_O

^b^Assessed by electron microscopy (SEM, TEM)

^c^Measured by Brunauer–Emmett–Teller (BET) method using Kr or N_2_ depending on the expected SSA

### Cell culture and particle exposure

J774 murine macrophages (ATCC#TIB-67) were grown to pre-confluence in Dulbecco’s modified Eagle Medium (DMEM) GlutaMAX supplemented with 10% fetal bovine serum (FBS), penicillin (100 U/ml) and streptomycin (100 µg/ml) (Invitrogen, Belgium). Primary mouse bone marrow derived cells obtained from the femurs of C57BL/6 mice and GSDMD-deficient mice (Heilig et al. 2018; Huot-Marchand et al. 2022) were differentiated in M1 macrophages by culturing in DMEM cell medium + 10% decomplemented FBS supplemented with 1% antibiotic–antimycotic, upon a 7 days treatment with 50 µg/ml granulocyte colony-stimulating factor (GM-CSF). This method refers to the procedure described by Oliveira & coworkers (Oliveira et al. 2003). Before particle exposure, J774 and primary macrophages were seeded in 96-well plates (50,000 cells/well) in DMEM GlutaMAX supplemented with penicillin (100 U/ml) and streptomycin (100 µg/ml) and allowed to adhere for 24 h at 37 °C in 5% CO_2_ atmosphere. Particles were heat-sterilized at 200 °C for 2 h prior to suspension to inactivate any trace of endotoxin. Silica suspensions were prepared just before use in serum free DMEM GlutaMAX and sonicated in a bath for 2 min (total delivered energy 3.6 kJ). Silica suspensions or serum-free DMEM GlutaMAX (negative control) were distributed in six to four replicates in the plates to the final concentrations of 1, 5, 10, 20, and 40 cm^2^/ml and incubated for 1, 3, 6, 18 or 24 h, depending on the investigated outcome. Comparable experimental conditions have been used in previous investigations (Leinardi et al. 2020; Turci et al. 2016).

In the experiments with inhibitors, particle exposure was carried out treating the cells with: anti-MARCO antibodies (0.1 μg/ml) (Bio-Rad, Hercules, CA, USA), catalase (100 U/ml), cytochalasin D (2.5 μg/ml), dextran sulfate (100 μg/ml), fucoidan (500 μg/ml), mannitol (50 mM), n-acetyl-cysteine (NAC, 0.1 mM), necrosulfonamide (NSA, 5 μM), poly-inosinic acid (poly-I, 50 µg/ml), and TAK242 (5 μM). All reagents were purchased from Sigma Aldrich (St. Louis, MI, USA), unless stated otherwise.

### Assessment of cell stress and cytotoxicity

The impact of particles on macrophage viability was evaluated by measuring the mitochondrial activity using the cell proliferation reagent WST1 (5% WST1 reagent in DMEM) (Roche Applied Science, Belgium) (Berridge and Tan 1998; Pavan et al. 2014). Plasma membrane lysis following particle cytotoxicity was evaluated by measuring extracellular LDH content via lactate dehydrogenase (LDH) release assay (Lison et al. 2008) using an LDH-Glo Cytotoxicity Assay kit (Promega, WI, USA), following manufacturer’s guidelines. Absorbance at 440 nm (WST-1) and luminescence (LDH) were monitored using a SpectraMax iD3 (Molecular Devices, CA, USA) microplate reader.

### Determination of IL-1α

IL-1α produced and accumulated into cells or released into culture supernatant was measured by enzyme-linked immunosorbent assay (ELISA). For intracellular IL-1α measurement, pelleted cells were lysed with 0.1% Triton X-100 in DPBS. ELISA kit for mouse IL-1α (DY400, detection limit 5 pg/ml) was used following manufacturer’s instructions (R&D Systems, Wiesbaden-Nordenstadt, Germany). Absorbance was determined using a SpectraMax iD3 (Molecular Devices, CA, USA) microplate reader set to 450 nm, with wavelength correction set to 540 nm.

### RNA extraction and quantification

IL-1α gene expression was quantified via quantitative real time polymerase chain reaction (qRT-PCR). Briefly, total RNA was extracted using TriPure isolation reagent (Invitrogen) according to manufacturer's instructions. Extracted RNA was reverse transcribed using random hexamers and Moloney Murine Leukemia Virus (M-MLV) reverse transcriptase (Invitrogen). Synthesized cDNA was amplified by qRT-PCR using a SYBR Green PCR Master Mix (Applied Biosystems, Life Technologies Corporation, CA, USA) on a StepOnePlus Real-Time PCR System (Applied Biosystems). Sequences of interest were amplified by using as forward primers (Invitrogen): CGG CTA CCA CAT CCA AGC AA (mouse 18S rRNA) and TTG AAG ACC TAA AGA ACT GTT ACA GTG AA (mouse IL-1α); as reverse primers: ATA CGC TAT TGG AGC TGG ATT ACC (mouse 18S rRNA) and GCC ATA GCT TGC ATC ATA GAA GG (mouse IL-1α). Gene expression of the house-keeping gene 18 s rRNA was used for normalization (Rabolli et al. 2014).

### Statistical analysis

Data are presented as mean ± standard error of the mean (SEM). Statistical analysis was performed with GraphPad Prism version 9.1 (GraphPad Software, La Jolla, California, USA). Differences among groups were analysed by one-way analysis of variance (ANOVA) followed by post hoc test as appropriate. Differences with *P* value < 0.0332 were considered statistically significant.

## Results

### Crystalline silica exposure triggers intracellular accumulation and extracellular release of IL-1α in a time- and dose-dependent manner

**Fig. 1:**
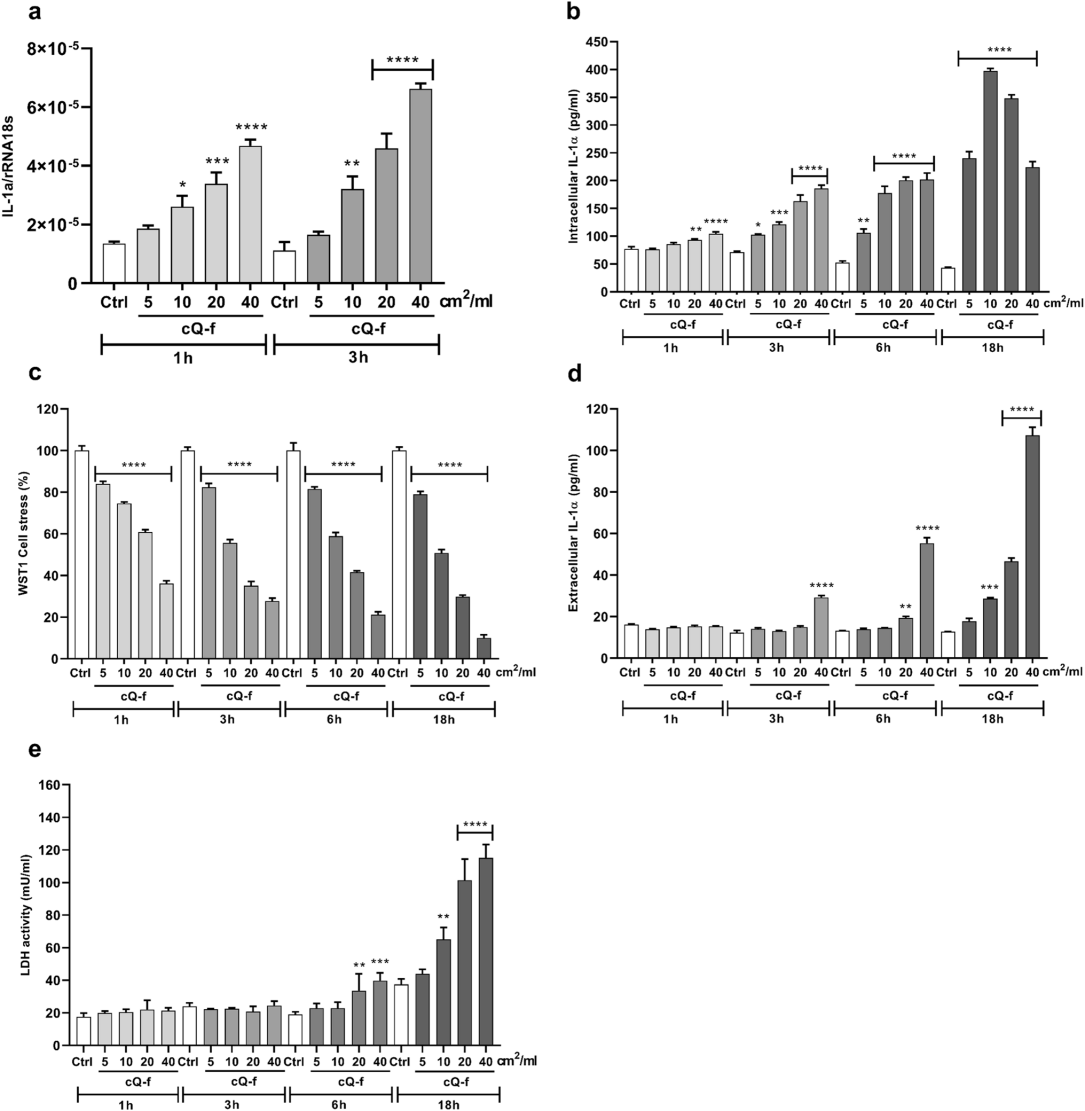
Early silica-induced IL-1α intracellular accumulation is followed by IL-1α extracellular release. Kinetics of IL-1α gene expression (**a**), intracellular IL-1α accumulation (**b**), metabolic cell stress evaluation (**c**), IL-1α extracellular release (**d**), and plasma membrane lysis (**e**) in J774 macrophages exposed to quartz particles. Cells were incubated with increasing concentrations (5, 10, 20, 40 cm^2^/ml) of quartz (cQ-f) for 1, 3, 6 and 18 h. IL-1α gene expression in macrophages was quantified by qRT-PCR (**a**), intracellular levels of IL-1α protein (**b**) and IL-1α released into culture supernatants (**c**) by ELISA, cell stress by WST1 assay (**d**) and plasma membrane lysis via evaluation of LDH leakage (**e**). Results of cell stress (**d**) are expressed as percentage of the control not exposed to particles (Ctl). Determinations were performed in quadruplicate and expressed as the mean ± SEM. Data from one representative experiment out of three, which show the same trends, are depicted. Differences between the control not exposed to particles (Ctl) and silica-exposed cells were evaluated with one-way ANOVA and Dunnett’s post hoc test. *P < 0.0332, **P < 0.0021, ***P < 0.0002 and ****P < 0.0001 *vs* Ctrl in each group. Horizontal bars above different columns combine comparable *P* values *vs* Ctrl

To investigate the mechanism of IL-1α regulation in macrophages following crystalline silica exposure, IL-1α gene expression (Fig. 1a) and intracellular protein accumulation (Fig. 1b) were first monitored. Murine J774 macrophages were exposed to increasing doses (5, 10, 20, and 40 cm^2^/ml) of a reference crystalline quartz (cQ-f,) at increasing time points (gene expression, RT-qPCR: 1 and 3 h; protein intracellular accumulation, ELISA: 1, 3, 6 and 18 h). Already 1 h after particle exposure, early IL-1α transcript and intracellular protein contents were increased in a dose-dependent manner with respect to control group (Fig. 1a and 1b). This revealed the very prompt ability of macrophages to sense quartz particles and respond by overexpressing IL-1α gene and stocking IL-1α protein. By monitoring the cell metabolic activity (WST1 assay), we evaluated whether IL-1α intracellular accumulation in macrophages was related to silica-induced cell stress. Early (already in 1 h) and dose-dependent impact of silica was observed on cell viability (Fig. 1c), suggesting that silica-induced early IL-1α upregulation and accumulation are initiated as soon as cell viability is challenged. At 18 h, the intracellular IL-1α protein levels at the highest doses of quartz (20 and 40 cm^2^/ml) were reduced with respect to 10 cm^2^/ml, suggesting that IL-1α was partially released in the extracellular medium (Fig. 1b). Indeed, we observed time- and dose-dependent late release of IL-1α in the extracellular milieu (supernatants, ELISA) (Fig. 1d), which was delayed with respect to the early intracellular accumulation. Extracellular LDH activity, as an outcome of plasma membrane lysis and necrosis (Chan et al. 2013), was also monitored (Fig. 1e). The LDH release was comparable to that of IL-1α release in culture supernatants (Fig. 1d), indicating that IL-1α release, necrosis and plasma membrane lysis were concomitant events in silica-exposed macrophages.

### IL-1α intracellular accumulation requires particle internalization

**Fig. 2:**
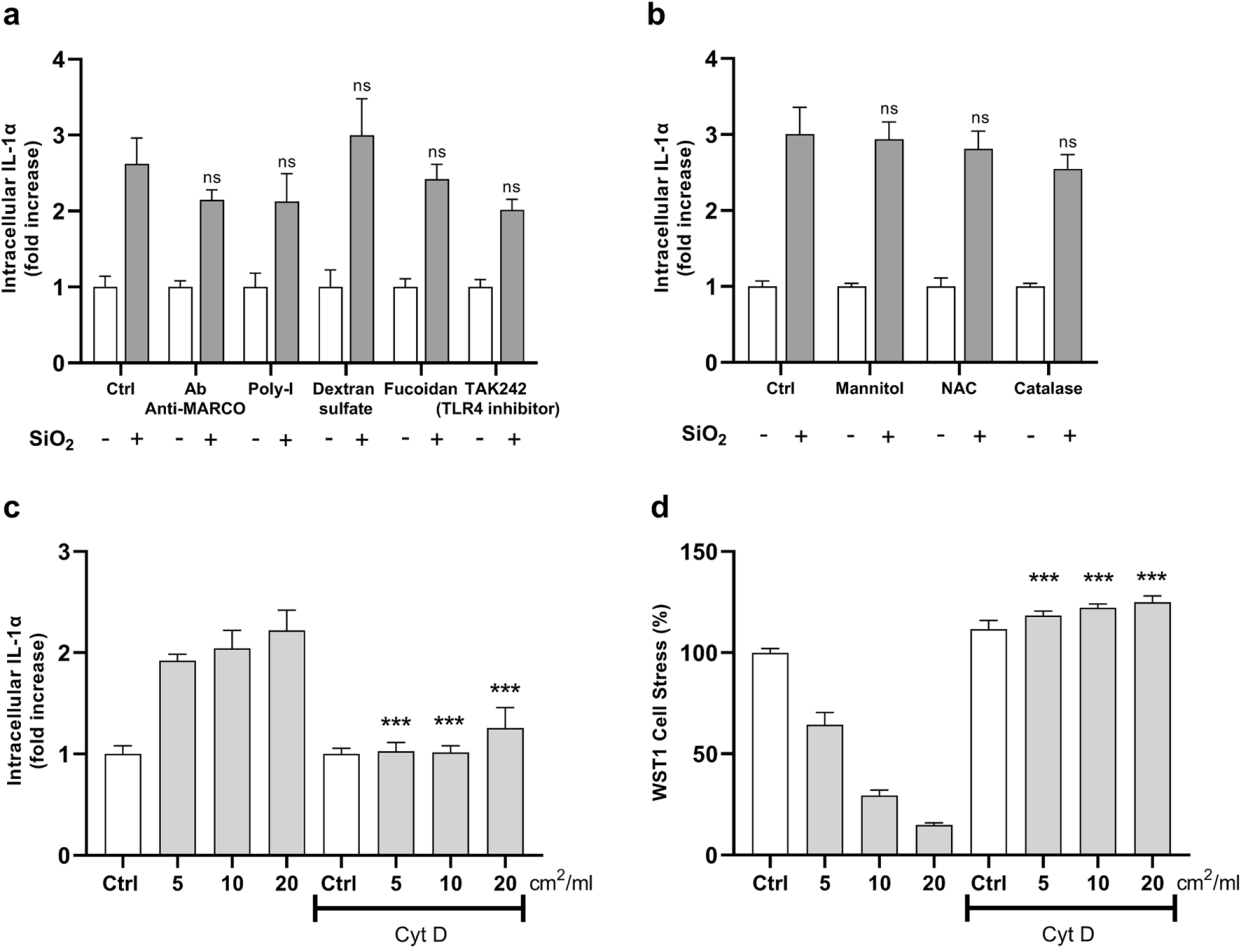
IL-1α intracellular accumulation is not related to innate receptor sensing or ROS cascade but requires particle internalization. Intracellular IL-1α accumulation (**a, b** and** c**), and cell stress (**d**) in J774 macrophages exposed to quartz particles in presence of selected inhibitors. Cells were incubated with cQ-f (20 cm^2^/ml) alone or by adding scavenger receptors (SR) and TLR4 inhibitors (**a**), or ROS scavengers (**b**). Effect of cytochalasin D (Cyt D) treatment and particle internalization on the intracellular accumulation of IL-1α (**c**) and cell stress (**d**) in J774 macrophages exposed 3 h to increasing concentrations of cQ-f (5, 10, and 20 cm^2^/ml). Data from one representative experiment out of three, which show the same trends, are depicted. Determinations were performed in quadruplicate and expressed as the mean ± SEM. Differences between groups treated only with silica and groups treated with silica + inhibitors, for the same dose of silica, were evaluated with one-way ANOVA, followed by Tukey’s multiple comparison test. *P < 0.0332, **P < 0.0021, ***P < 0.0002 and ****P < 0.0001, Ctrl *vs* treatment with inhibitors

We first explored the mechanism of IL-1α intracellular accumulation following particle exposure by targeting innate receptors (scavenger receptors, SR; toll-like receptor 4, TLR4), and reactive oxygen species (ROS) cascade. SR sensing and intracellular ROS pathways contribute to silica recognition and subsequent macrophage activation leading to the release of pro-inflammatory cytokines and chemokines (Nishijima et al. 2017; Tsugita et al. 2017). SR inhibitors included an anti-SR-A6 (MARCO) antibody (Beamer et al. 2016), a SR-B1 antagonist (polyinosinic acid, poly-I) (Tsugita et al. 2017), and two competitive inhibitors of SR-A (dextran sulfate and Fucoidan) (Platt et al. 1996; Segers et al. 2012). TAK242 was used to prevent TLR4 activation (Chan et al. 2018). The role of ROS was investigated by using three scavengers: a hydroxyl radical (OH^•^) scavenger (i.e., mannitol) (Scarfi et al. 2009), a synthetic precursor of intracellular cysteine and glutathione (n-acetyl-cysteine, NAC) (Sun 2010), and a key antioxidant enzyme (catalase) (Scarcello et al. 2020). None of the SR, TLR4 or ROS inhibitors significantly reduced IL-1α intracellular accumulation induced by silica particles (Fig. 2a and b, respectively).

We then assessed whether IL-1α accumulation was dependent on particle internalization by macrophages (Pavan et al. 2014). Cytochalasin D (Cyt D), an inhibitor of actin filament polymerization, was used to prevent phagocytosis. Cyt D treatment prevented IL-1α intracellular accumulation 3 h after silica exposure (Fig. 2c). As expected, cytotoxicity/cell stress induced by crystalline silica was also prevented (Fig. 2d), confirming that cQ-f particles were not internalized by macrophages after Cyt D treatment. Overall, these results indicate that particle internalization is required to promote IL-1α intracellular accumulation.

### IL-1α intracellular accumulation following particle internalization is unrelated to physico-chemical features

**Fig. 3:**
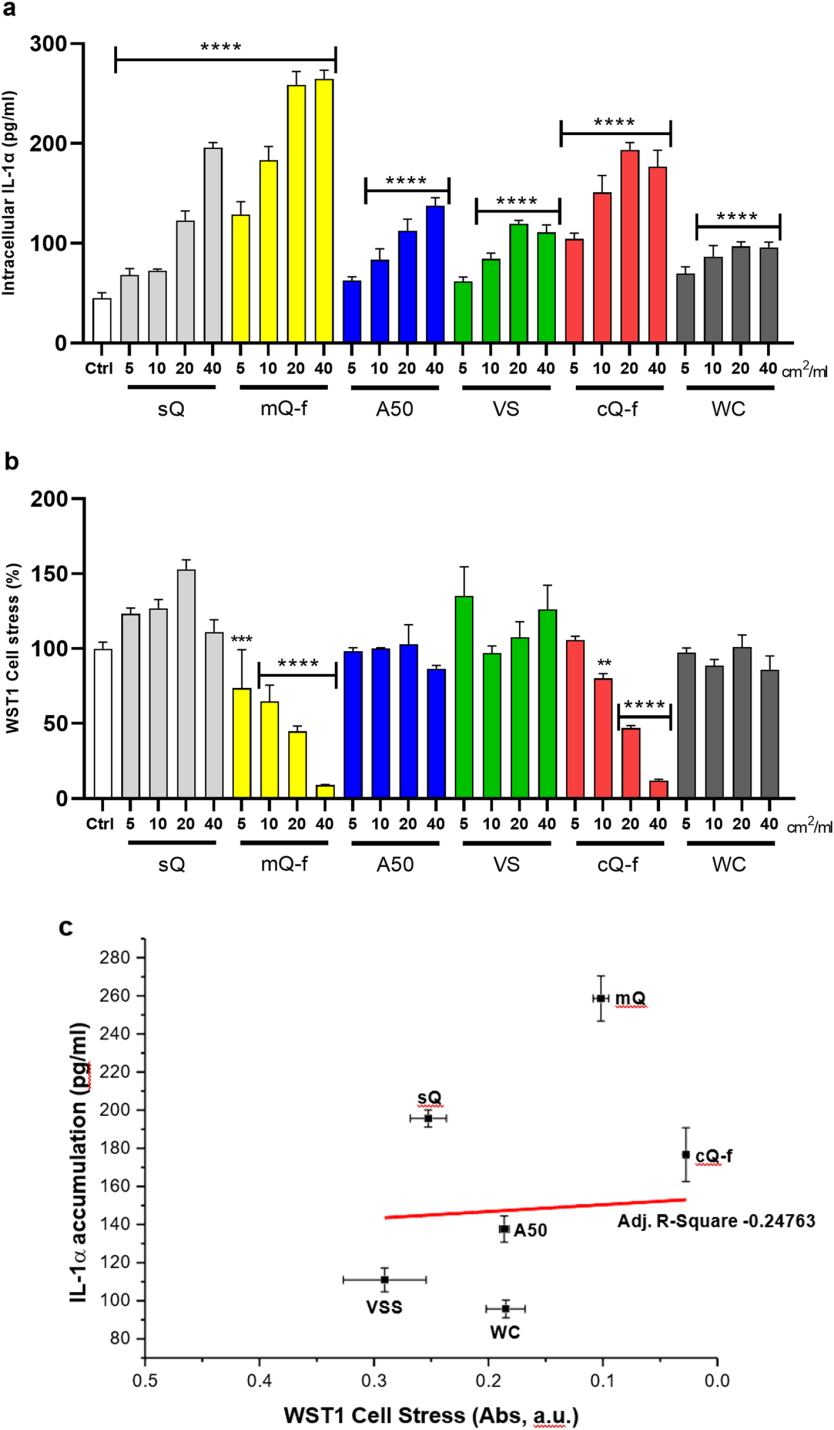
IL-1α is accumulated intracellularly regardless of macrophage stress and particle physico-chemical features. Intracellular IL-1α accumulation (**a**), cell stress (**b**), and correlation between cell stress (WST1) and IL-1α accumulation (**c**) in J774 macrophages exposed to amorphous and crystalline silica particles with different physico-chemical properties (40 cm^2^/ml, 3 h). Cells were incubated 3 h with increasing concentrations (5, 10, 20, 40 cm^2^/ml) of particles. WC particles were used as negative reference particle. Intracellular levels of IL-1α protein were quantified by ELISA (pg/ml) and cell stress via WST1 assay. Results of cell stress are expressed as percentage of the control not exposed to particles (Ctrl) (**b**) and Absorbance units (**c**). Lower Abs values indicate higher cell stress. Determinations were performed in quadruplicate and expressed as the mean ± SEM. Data from one representative experiment out of three, which show the same trends, are depicted. Differences between the control not exposed to particles (Ctrl) and quartz-exposed cells were evaluated with one-way ANOVA and Dunnett’s post hoc test. *P < 0.0332, **P < 0.0021, ***P < 0.0002 and ****P < 0.0001 vs Ctrl. Horizontal bars above different columns combine comparable *P* values vs Ctrl

To assess whether the early IL-1α intracellular accumulation following silica exposure was related to specific particle characteristics, including NFS content, J774 macrophages were incubated with silica particles characterized by diverse physico-chemical features (Table 1). The set of crystalline particles included two NFS-rich fractured quartz (mQ-f, cQ-f), found to be cytotoxic and membranolytic in a previous investigation (Pavan et al. 2020), and a NFS-poor synthetic quartz (sQ), known to be biologically inert in toxicological studies (Leinardi et al. 2020; Pavan et al. 2020; Turci et al. 2016). Two amorphous specimens were also studied: a fumed silica (A50) previously showing cytotoxicity and inflammogenicity (Gazzano et al. 2012; Zhang et al. 2012), and a vitreous silica (VS) cytotoxic in vitro (Ghiazza et al. 2010). Non-cytotoxic and non-inflammogenic tungsten carbide (WC) particles (Huaux et al. 1999) were used as negative reference sample. IL-1α intracellular accumulation and cell stress (3 h) were investigated in J774 macrophages exposed to increasing concentrations of particles (5, 10, 20 and 40 cm^2^/ml) (Fig. 3). All the investigated samples, including non-cytotoxic WC and sQ, induced an early and dose-dependent accumulation of IL-1α, not related to their specific impact on cell stress (Fig. 3a and b). Indeed, no correlation between impairment of cellular metabolism (WST1, absorbance units) and IL-1α accumulation was observed (Fig. 3c). This result suggested that particles activated IL-1α production and accumulation process regardless of cell stress and surface properties, including NFS content. Overall, these findings indicate that macrophages rapidly respond to inert and cytotoxic particles by stocking IL-1α in the intracellular compartment.

### GSDMD pores are the exclusive channel regulating IL-1α extracellular release from necrotic macrophages after silica exposure

**Fig. 4:**
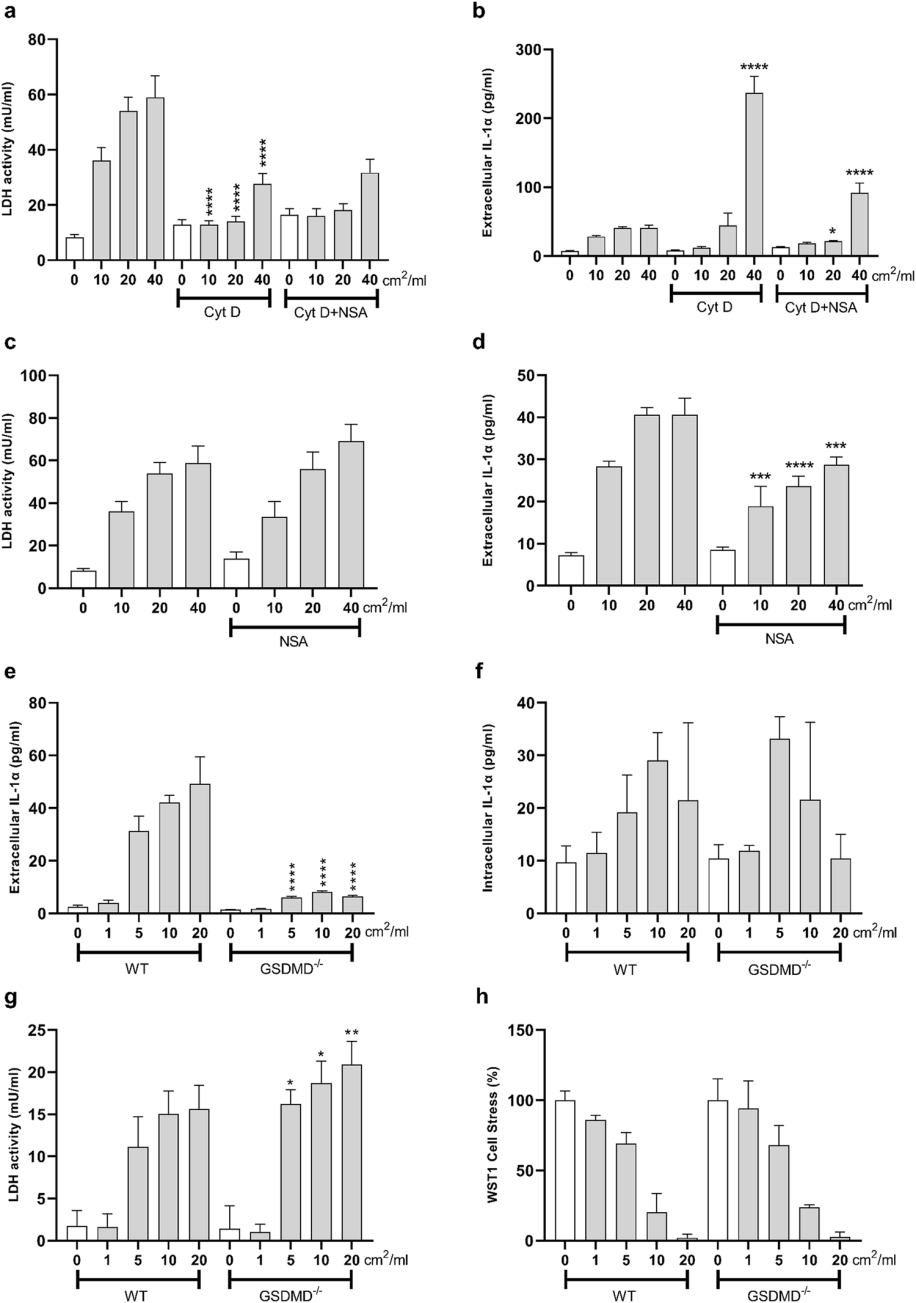
GSDMD pores regulate IL-1α extracellular secretion by silica-exposed macrophages. Plasma membrane lysis (**a, c, g**), extracellular IL-1α release (**b, d, e**), intracellular accumulation (**f**) and cell stress (**h**) from J774 macrophages, and wild-type (WT) and GSDMD-deficient (GSDMD^−/−^) primary macrophages exposed to crystalline silica. Depending on the outcome, cells were incubated 3 or 24 h with increasing concentrations of cQ-f (J774 macrophages: 5, 10, 20, 40 cm^2^/ml; primary macrophages: 1, 5, 10, 20 cm^2^/ml). J774 exposure was carried out in presence of cytochalasin D alone (Cyt D), cytochalasin D + NSA (Cyt D + NSA), and NSA alone (NSA). Plasma membrane lysis (24 h) was evaluated by extracellular LDH activity (mU/ml) (**a, c, f**). Extracellular (24 h) and intracellular (3 h) levels of IL-1α were quantified by ELISA (pg/ml) (**b, d, e, g**), and cell stress (3 h) via WST1 assay (**h**). Determinations were performed in quadruplicate and expressed as the mean ± SEM. Data from one representative experiment out of three, which show the same trends, are depicted. Differences between groups treated only with silica and groups treated with silica + Cyt D and/or NSA, for the same dose of silica, were evaluated with one-way ANOVA, followed by Tukey’s multiple comparison test. *P < 0.0332, **P < 0.0021, ***P < 0.0002 and ****P < 0.0001

We then clarified the exact mechanism of subsequent IL-1α release from macrophages following silica exposure, which was concomitant to LDH release and necrosis (Fig. 1, 18 h). To test plasma membrane lysis, Cyt D-pre-treated J774 macrophages were exposed to cQ-f silica. As expected, Cyt D dramatically decreased LDH leakage from J774 macrophages incubated with different doses of silica (Fig. 4a). On the contrary, IL-1α release was not significantly altered by Cyt D treatment in macrophage cultures challenged with 10 and 20 cm^2^/ml of silica. At the dose of 40 cm^2^/ml, IL-1α release was, in contrast, exacerbated following Cyt D treatment (Fig. 4b). These data strongly suggested that, besides the simple passive release taking place via plasma membrane lysis, additional mechanisms were implicated in the release of IL-1α by necrotic macrophages. GSDMD is an essential membrane pore for IL-1α secretion by bone-marrow derived macrophages (BMDM) after lipopolysaccharide and nigericin treatment (Tsuchiya et al. 2021). We then used NSA, a chemical inhibitor of GSDMD, to block GSDMD activity (Rathkey et al. 2018; Sborgi et al. 2016). The addition of NSA to cytochalasin D-treated cells (Cyt D + NSA) reduced the release of IL-1α at 40 cm^2^/ml (Fig. 4b), without further modulating LDH (Fig. 4a). We additionally evaluated the effect of NSA alone in silica-exposed macrophages. While LDH was not affected (Fig. 4c), NSA reduced IL-1α release as compared to the same dose of quartz alone (Fig. 4d). Similarly, in GSDMD-deficient macrophages obtained from bone marrow progenitors of deficient mice, IL-1α extracellular secretion after particle exposure (24 h) was dramatically lower when compared to silica-exposed wild-type (WT) macrophages (Fig. 4e). Interestingly, this decrease was observed despite elevated early (3 h) IL-1α intracellular accumulation (Fig. 4f). As noticed with the GSDMD inhibitor NSA, GSDMD deficiency did not reduced LDH release (Fig. 4g), indicating that IL-1α and LDH were released via different pathways. Early cell stress evaluation suggested that WT and GSDMD^–/–^ primary macrophages were comparably sensitive to crystalline silica exposure (Fig. 4h). Overall, these observations demonstrate that silica-induced IL-1α secretion from necrotic macrophages is a process mainly orchestrated by the pore-forming GSDMD, not merely requiring complete plasma membrane lysis.

### IL-1α extracellular secretion is triggered by cytotoxic NFS-rich silicas

**Fig. 5:**
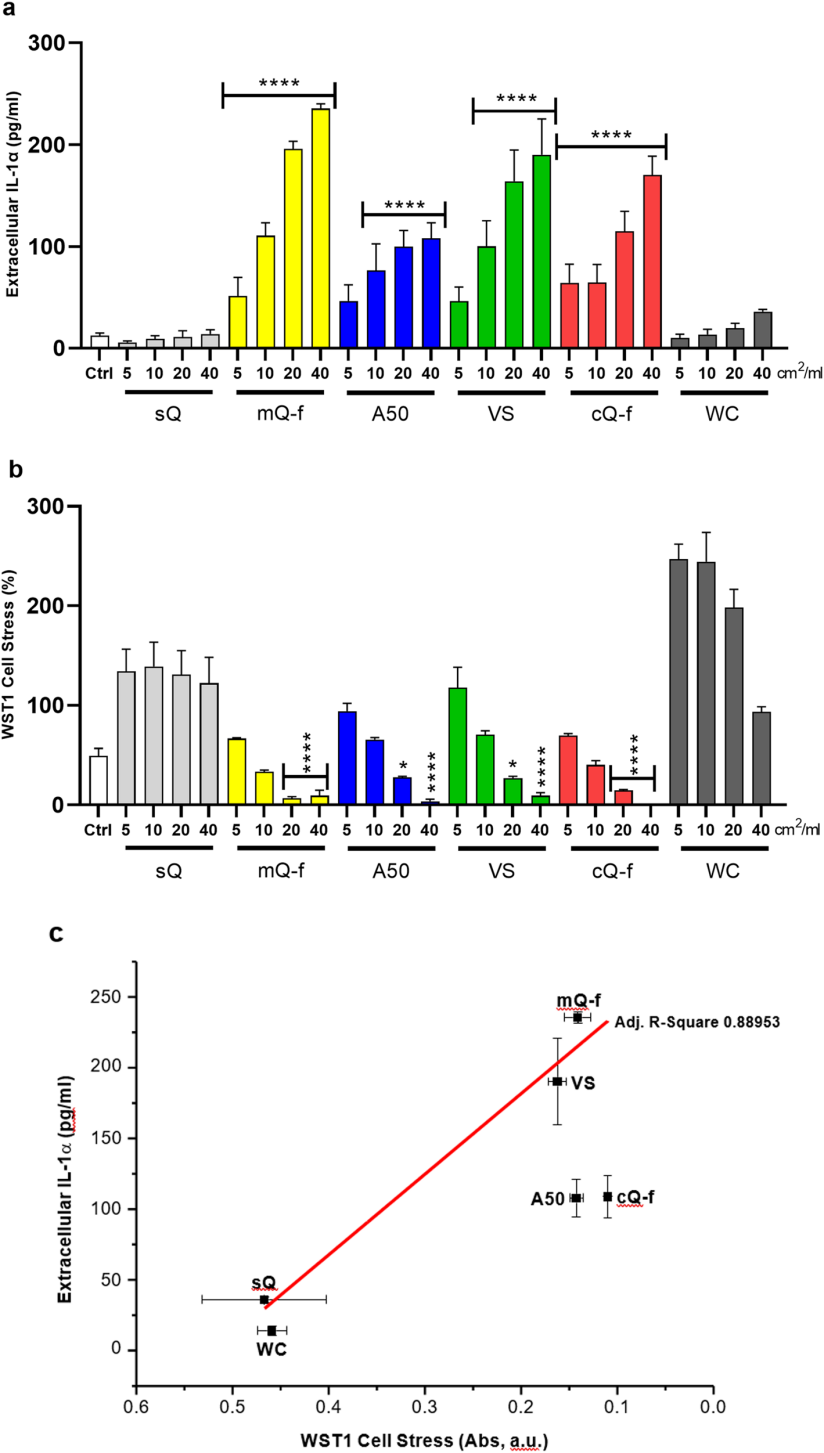
IL-1α secretion is triggered by cytotoxic silica particles impacting cell viability. Extracellular IL-1α secretion (**a**), cell stress (**b**), and correlation between cell stress (WST1) and extracellular secreted IL-1α (**c**) in J774 macrophages exposed to amorphous and crystalline silica particles with different physico-chemical properties (40 cm^2^/ml, 24 h). Cells were incubated 24 h with increasing concentrations (5, 10, 20, 40 cm^2^/ml) of particles. WC particles were used as negative reference particle. Intracellular levels of IL-1α protein were quantified by ELISA (pg/ml) and cell stress via WST1 assay. Results of cell stress are expressed as percentage of the control not exposed to particles (Ctrl) (**b**) and Absorbance units (**c**). Lower Abs values indicate higher cell stress. Determinations were performed in quadruplicate and expressed as the mean ± SEM. Data from one representative experiment out of three, which show the same trends, are depicted. Differences between the control not exposed to particles (Ctrl) and quartz-exposed cells were evaluated with one-way ANOVA and Dunnett’s post hoc test. *P < 0.0332, **P < 0.0021, ***P < 0.0002 and ****P < 0.0001 vs Ctrl. Horizontal bars above different columns combine comparable *P* values vs Ctrl

We then refined the relationship between the IL-1α extracellular active secretion and cytotoxic activity of silica by exposing macrophages to particles having different NFS content. Data showed in Fig. 5 reveal that only particles able to strongly impact cell viability (WST1, 24 h) (i.e., mQ-f, A50, VS and cQ-f), triggered significant dose-dependent IL-1α extracellular secretion. This effect was likely related to NFS occurrence, corroborating previous findings (Turci et al. 2016; Leinardi et al. 2020; Pavan et al. 2020). Indeed, the NFS-rich mQ-f, cQ-f, and the two amorphous silicas, induced both IL-1α secretion and cytotoxicity (Fig. 5a and b). In contrast, IL-1α extracellular secretion and cell stress were not affected after NFS-poor synthetic silica (sQ) and WC exposure at any investigated dose. A linear regression analysis between WST1 (absorbance units) and IL-1α (40 cm^2^/ml of particle/well) confirmed the relationship between particle-induced cytotoxicity and IL-1α secretion (Fig. 5c), supporting the role of NFS in IL-1α active secretion.

Overall, these findings indicate that, even though IL-1α intracellular stock is quickly amplified by inert and cytotoxic NFS-rich particles (Fig. 3), only cytotoxic silicas affecting cell viability activate GSDMD-orchestrated IL-1α secretion from necrotic macrophages (Fig. 5).

## Discussion

After cellular insults, the release of self-intracellular components (known as damage-associated molecular patterns, DAMPs) activates innate and adaptive immunety and promotes pathological responses (Gong et al. 2020). DAMPs are secreted via specific active mechanisms, or after plasma membrane disruption upon cell injury (Leinardi et al. 2022). IL-1α is a paramount DAMP released in the extracellular environment and implicated in the production of cytokines and chemokines inducing monocyte and neutrophil influx in the injury site (Dinarello 2018). In a previous work, we demonstrated that IL-1α was released from existing stocks in alveolar macrophages after crystalline silica exposure. This process promoted in vivo lung inflammation by triggering IL-1β activation and release (Rabolli et al. 2014). In accordance with these observations, it has been demonstrated that prolonged lung IL-1α secretion from silica-exposed alveolar macrophages also exacerbated allergic responses (Kuroda et al. 2016). Similar outcomes including IL-1α release and robust lung inflammation were noticed in silica-treated lupus-prone mice (Chauhan et al. 2021). Beside these convincing findings connecting IL-1α and silica-induced chronic pathologies, no investigation has been carried out on the molecular mechanisms driving IL-1α release by silica-exposed macrophages.

**Fig. 6:**
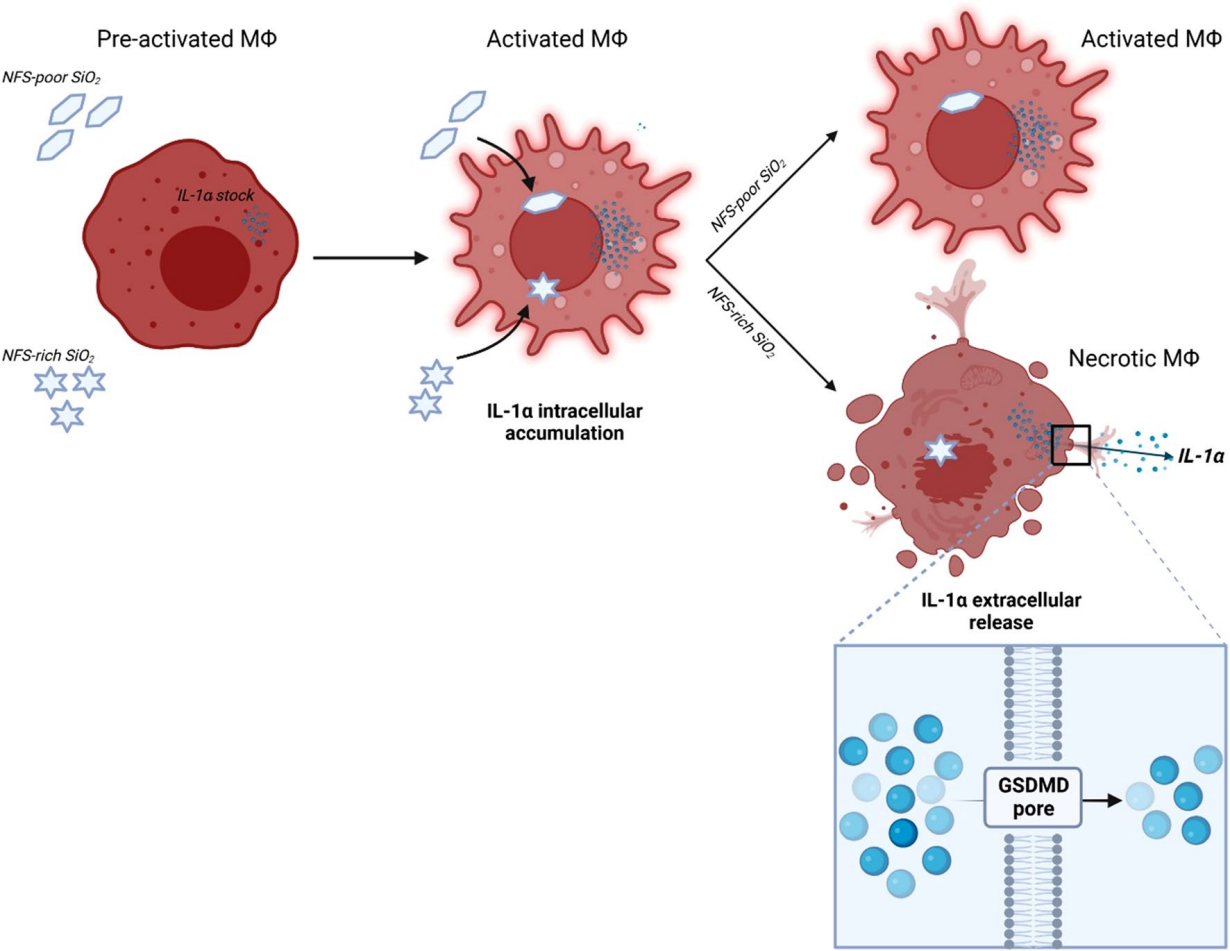
GSDMD-pores formation modulate IL-1α secretion from silica-exposed necrotic macrophages. Independently from NFS content, silica internalization induces macrophage activation enhancing IL-1α intracellular stock by upregulating IL-1α gene transcription, leading to protein accumulation. Following IL-1α accumulation, the exposure to NFS-rich cytotoxic silica leads to the formation of GSDMD pores in the plasma membrane of necrotic macrophages, orchestrating active IL-1α extracellular secretion

In this study, we observed that the pre-existing stock of IL-1α in macrophages was further increased via the prompt induction of IL-1α gene expression and protein accumulation after particle exposure. This effect solely depended on particle internalization and was not related to cell activation or stress. This observation thus supports the novel notion that particle uptake is sufficient to activate macrophages by increasing cellular stocks of IL-1α. It is known that IL-1α upregulation is activated by several stimuli comprising TLR agonists or oxidative stress but also IL-1α itself. Because in our investigation the use of TLR4 inhibitors and ROS scavengers did not impact IL-1α intracellular accumulation, we speculate that the pre-existing membrane-bound IL-1α interacts with membrane interleukin-1 receptor 1 (IL-1R1) during particle internalization process. As already observed by Di Paolo and colleagues, this interplay induce a biochemical cascade triggering NFκB activation, IL-1α expression and protein intracellular accumulation (Di Paolo and Shayakhmetov 2016). Contrariwise, the extracellular release of IL-1α is more finely regulated and specifically depends on particle features. Indeed, the release of stocked IL-1α only occurred when macrophages were exposed to NFS-rich reactive silica and underwent necrosis. Interestingly, these observations suggest that the mechanism regulating IL-1α secretion in silica-exposed macrophages comprises a two-hit process: (i) the rapid IL-1α intracellular accumulation after particle internalization by sensitized macrophages, and (ii) the subsequent IL-1α secretion exclusively from macrophages undergoing necrosis upon exposure to NFS-rich silica (Fig. 6). We demonstrated that GSDMD pores were necessary for IL-1α secretion from macrophages exposed to silica. GSDMD mediates pyroptotic regulated cell death after being cleaved by caspase-1. Pyroptosis is a regulated modality of necrotic cell death orchestrating innate immunity and inflammation (Man et al. 2017). Upon caspase-1 activation, GSDMD pores are formed through a coordinated process where the N-terminal fragment of GSDMD (GSDMD p30) targets the plasma membrane, allowing permeabilization and leading to pyroptosis (Sborgi et al. 2016). GSDMD pores possess an inner diameter of 15 nm and trigger water and Ca^2+^ ions influx, which activate calpain, cell swelling and plasma membrane lysis (Xia et al. 2021). The crucial role played by GSDMD in guiding the pro-inflammatory effect of silica observed in our in vitro study is in line with previous studies in GSDMD-deficient primary macrophages treated with particulate- (monosodium urate—MSU—crystals, and alum) and non-particulate- (LPS and nigericin) inflammasome activators (Evavold et al. 2018; Gross et al. 2012; Tsuchiya et al. 2021). Also, our data are supported by recent in vivo investigations carried out in GSDMD-deficient mice, which, contrary to WT animals, were resistant to develop lung silicosis after silica instillation (Benmerzoug et al. 2018; Song et al. 2022). Similarly, several reports show that IL-1α depletion reduces lung inflammatory responses (Cassel et al. 2008; Guo et al. 2013; Rabolli et al. 2014), suggesting that the intracellular pyroptotic machinery, including GSDMD and IL-1α, is crucial in the pathogenesis of silica-induced lung inflammation. Thus, our data clarify how macrophages actively orchestrate the secretion of a key DAMP upon entering in contact with cytotoxic and pro-inflammatory silica.

We noticed that GSDMD-deficient primary macrophages exposed to crystalline silica also encountered plasma membrane impairment. The pore-independent secretion of LDH in necrotic GSDMD^−/−^ and NSA-treated macrophages further supported the specificity of IL-1α for GSDMD. This observation suggests that GSDMD inhibition or absence does not protect macrophages from silica-induced necrosis, corroborating what previously observed by Rathkey (Rathkey et al. 2018). Also, the dichotomy between silica-induced active IL-1α secretion and LDH release following plasma membrane lysis is supported by recent literature data demonstrating the critical role played by pore-forming proteins in regulated necrotic cell death (Flores-Romero et al. 2020; Wang and Shao 2021). The necrotic process leading to plasma membrane lysis in silica-exposed macrophages might be actively orchestrated by other gasdermins. GSDME is an additional member of the gasdermin family, found to be implicated in cytokine secretion and lytic necrosis in GSDMD-deficient cells (Zhou and Abbott 2021). Interestingly, LDH release was strongly inhibited in GSDME-deficient cells (Dong et al. 2022). GSDME involvement in silica-induced regulated necrosis and LDH release needs to be addressed in future investigations.

Our data demonstrated that cell stress-impacting fractured crystalline and amorphous silicas, whose surface is characterized by NFS occurrence (Pavan et al. 2020), triggered dose-dependent IL-1α secretion. Conversely, synthetic as-grown quartz (sQ) and WC were not active. sQ, because of NFS negligible amount, was unable to induce any detrimental in vitro effect, confirming what previously observed in different cell types (Leinardi et al. 2020; Pavan et al. 2020; Turci et al. 2016). The two amorphous silicas, i.e. pyrogenic (A50) and vitreous (VS) particles, induced IL-1α accumulation and secretion in J774 macrophages. These observations agree with previous findings showing that some types of amorphous (nano)silica might potentially induce pulmonary inflammation (Croissant et al. 2020; Leinardi et al. 2022), and suggest that the detailed examination of the surface properties is critical for predicting the in vitro cytotoxic and inflammatory potential of silica particles.

## Conclusions

Our findings unveil novel insights on the molecular mechanisms leading to DAMP release from necrotic macrophages, a process driving the inflammatory activity of NFS rich-silica. GSDMD pore formation in the macrophage membrane explains IL-1α secretion from existing and enhanced intracellular stocks (Fig. 6). This pore-guided IL-1α secretion is an active and well-regulated process, which overcomes the theory proposing a passive and unregulated release of IL-1α accompanying macrophage membrane lysis. Our results also suggest that the detailed investigation of the surface physico-chemical properties of particles, supported by the evaluation of the in vitro IL-1α extracellular content and GSDMD-pore formation, represents a convenient approach to assess and predict the pulmonary hazard of silica particles and other types of particulate matter. Finally, the dynamic and finely orchestrated IL-1α secretion represents an additional and critical first event in the AOP underlying silica inflammogenicity previously proposed (Pavan and Fubini 2017).

## Data Availability

The dataset used and/or analysed during the current study are available from the corresponding author on reasonable request.

## Abbreviations

AM: Alveolar macrophages
AOP: Adverse outcome pathway
BET: Brunauer–Emmett–Teller
BMDM: Bone marrow derived macrophages
DAMP: Damage-associated molecular patterns
DMEM: Dulbecco’s modified eagle medium
ELISA: Enzyme-linked immunosorbent assay
FBS: Fetal bovine serum
FPIA: Flow particle image analysis
GMCSF: Granulocyte macrophage colony-stimulating factor
GSDMD: Gasdermin D
GSDME: Gasdermin E
IARC: International agency for research on cancer
LMP: Lysosome membrane permeabilization
LDH: Lactate dehydrogenase
MARCO: Macrophage receptor with collagenous structure
MIE: Molecular initiating event
NFκB: Nuclear factor kappa-light-chain-enhancer of activated B cells
NFS: Nearly free silanols
NLS: Nuclear localization sequence
RCS: Respirable crystalline silica
SEM: Scanning electron microscopy
SR: Scavenger receptors
SSA: Specific surface area
TEM: Transmission electron microscopy
TLR: Toll-like receptors
WST1: Water-soluble tetrazolium salt
WT: Wild-type
